# Association between numbing-spicy related dietary behavior and hyperuricemia in a Southwestern Chinese population

**DOI:** 10.3389/fnut.2026.1795146

**Published:** 2026-07-20

**Authors:** Ling Zhang, Huali Xiong, Feiqiang Ren

**Affiliations:** 1 Department of Urology, Chongqing Rongchang District Hospital of Traditional Chinese Medicine, Chongqing, China; 2 Department of Public Health, Center for Disease Control and Prevention of Rongchang District, Chongqing, China; 3 Department of Urology, Chongqing Hospital of Traditional Chinese Medicine, Chongqing, China

**Keywords:** China Multi-Ethnic Cohort Study, dietary pattern, hyperuricemia, numbing food intake, spicy food intake

## Abstract

**Background:**

The consumption of numbing–spicy foods is highly prevalent in southwestern China. However, existing studies examining the relationship between such dietary patterns and disease risk have primarily focused on spicy food intake, with limited attention given to the “numbing” component. This study therefore aimed to systematically investigate the association between both numbing and spicy food intake and hyperuricemia.

**Methods:**

All data were derived from the China Multi-Ethnic Cohort (CMEC) study in Rongchang district, Chongqing Municipality, a representative area of southwestern China with high prevalence of numbing-spicy food consumption. Information on numbing and spicy food intake were collected using a standardized questionnaire. The associations between numbing/spicy food consumption and hyperuricemia were evaluated using multivariable logistic regression models and restricted cubic spline analyses. Subgroup analyses were further conducted across diverse demographic and clinical characteristics. To assess potential combined relation, both multiplicative and additive interaction analyses were performed. In addition, mediation and sensitivity analyses were undertaken to assess the robustness of the findings.

**Results:**

A total of 2,265 participants aged 30–60 years were included in the analysis. The mean age was 45.21 years (SD = 7.84), and the overall prevalence of hyperuricemia was 13.20%. After adjustment for potential confounding factors, the combined intake of numbing and spicy foods were positively associated with hyperuricemia, with an odds ratio (OR) of 2.689 (95%CI: 2.045–3.536, *P* < 0.001). Restricted cubic spline analyses further indicated a nonlinear, positive relationship with hyperuricemia (*P*_*for overall*_ < 0.001, *P*_*for*_ _*nonlinear*_ = 0.020). These associations remained robust across subgroup and sensitivity analyses. No significant multiplicative interaction was detected, combined numbing-spicy intake was positively associated with hyperuricemia (*OR* = 4.703, 95%CI: 1.751–12.902) compared with neither intake in additive interaction. However, formal measures of additive interaction, including the relative excess risk due to interaction, attributable proportion, and synergy index, did not reach statistical significance. In exploratory mediation analyses, the poultry-aquatic dietary pattern statistically explained 6.58% of the association for spicy-only intake and 14.54% of the association for combined numbing and spicy intake. No significant mediation associations were identified for other dietary patterns.

**Conclusion:**

These findings contribute new evidence regarding the link between numbing–spicy food intake and hyperuricemia, extending prior research that has focused primarily on spicy food consumption alone. Accordingly, future investigations into the dietary etiology of hyperuricemia in southwestern China should move beyond treating spicy food intake as an isolated exposure and the joint effects of characteristic numbing–spicy dietary behaviors and their associated culinary context on the prevalence of hyperuricemia should be systematically considered.

## 1 Introduction

Hyperuricemia (HUA) is a metabolic disorder characterized by impaired renal and extrarenal uric acid excretion and abnormally elevated serum uric acid levels, and it has emerged as a significant and growing global public health challenge (1). From 1990 to 2021, the global number of incident gout cases increased substantially, rising from 1.94 million to 3.89 million (1). Gout is a metabolic disorder characterized by hyperuricemia and the deposition of monosodium urate crystals in tissues, leading to inflammation and subsequent tissue damage (2). Studies have indicated that hyperuricemia is associated with chronic kidney disease (3), hypertension (4), diabetes (5). Long-term hyperuricemia may also contribute to the development of atherosclerosis and increase the risk of cardiovascular disease (6).

In recent years, concomitant with socioeconomic development and lifestyle changes, the prevalence of hyperuricemia in the Chinese population has increased markedly. A meta-analysis reported that the overall prevalence of hyperuricemia in mainland China was 17.4%, with the prevalence among males being approximately twice that among females (22.7% vs. 11.0%) (7). The prevalence of hyperuricemia in China has shown a consistent upward trajectory over time, with a noticeable shift toward younger age groups. It has now become the second most common metabolic disorder in China, following diabetes (8). Previous studies had revealed that elevated blood pressure (9), fasting blood glucose (10), lipid profiles and waist circumference (10) were associated with hyperuricemia. In addition to disease-related factors, dietary patterns were also closely associated with hyperuricemia (11, 12), including alcoholic beverage intake (13), increased intake of livestock meat and poultry. Traditional dietary risk factors, as mentioned above, have been extensively studied. More recently, a growing body of research has focused on the association between trace elements and hyperuricemia, with evidence suggesting that long-term relatively high dietary manganese intake may reduce the risk of hyperuricemia (14) and high dietary retinol intake is correlated with increased hyperuricemia susceptibility (15). However, the association between hyperuricemia risk and specific, culturally distinct, and increasingly popular dietary patterns—such as the characteristic numbing–spicy food consumption prevalent in southwestern China—has not yet been systematically elucidated.

As one of the eight major cuisines of China, Sichuan cuisine is characterized by its distinctive numbing, spicy, savory, and aromatic flavors, with its core sensory attributes being “numbing” and “spicy” (16). Sichuan cuisine uniquely combines the tingling sensation of Sichuan peppercorns with the heat of chili peppers. Unlike Thai or Mexican spicy foods, which focus solely on capsaicin-induced heat, authentic mala balances the floral and citrus notes of huājiāo with the deep warmth of chili. This dual-sensation technique, documented over China’s 2,000-year culinary history, contributes to the complexity of Sichuan cuisine. Accordingly, our investigation emphasizes not capsaicin heat alone, but the potential combined health effects of this defined numbing–spicy complex. In Sichuan cuisine, common sources of the numbing sensation include Sichuan pepper oil, dried Sichuan pepper, and fresh Sichuan pepper, while the spicy taste is typically derived from chili sauce, chili oil, dried chili peppers, and fresh chili peppers.

Benefiting from the humid basin climate and the historical integration of diverse cultures via immigration, river ports, and salt-trade routes, the consumption of numbing and spicy foods has become a significant component of daily energy intake among residents in southwestern China (Sichuan, Chongqing, Guizhou, and Yunnan). Previous studies have reported that 88.13% of adults in this region consume spicy foods (17). The consumption of numbing–spicy foods is deeply embedded in the daily diets of residents in southwestern China, representing a highly prevalent dietary habit. However, studies have reported that the prevalence of hyperuricemia in this region is 21.2%, significantly higher than the national average of 16.4% (18). Although traditional risk factors for hyperuricemia are well recognized, they cannot fully explain the high burden of hyperuricemia observed in the southwestern Chinese population.

This study aimed to address this knowledge gap by focusing on residents of southwestern China in Rongchang District, which is located in the western part of Chongqing Municipality. As one of the core regions of Sichuan cuisine culture, this area is characterized by representative numbing-spicy dietary habits in southwestern China. This region was selected not only because of the high prevalence and representativeness of numbing–spicy food consumption but also because it offers an ideal natural population setting to investigate the potential impact of dietary culture on hyperuricemia risk. The study systematically assesses the association between numbing–spicy intake and the risk of hyperuricemia. The findings are expected to provide novel scientific evidence for understanding the role of dietary culture in the epidemiology of hyperuricemia and to inform the development of region-specific primary management strategies and dietary guidelines.

## 2 Materials and methods

### 2.1 Study design

This study utilized baseline data from the China Multi-Ethnic Cohort (CMEC) study (19) in Rongchang district. CMEC study was a large community-based initiative established by Sichuan University in southwestern China, which was conducted between September 2018 and January 2019. The baseline survey employed three-stage stratified random sampling method in Rongchang district, with detailed procedures reported elsewhere (20) The Sichuan University Ethics Committee approved this protocol (No. K2016038) and all participants provided written informed consent prior to enrollment.

### 2.2 Study population

**FIGURE 1:**
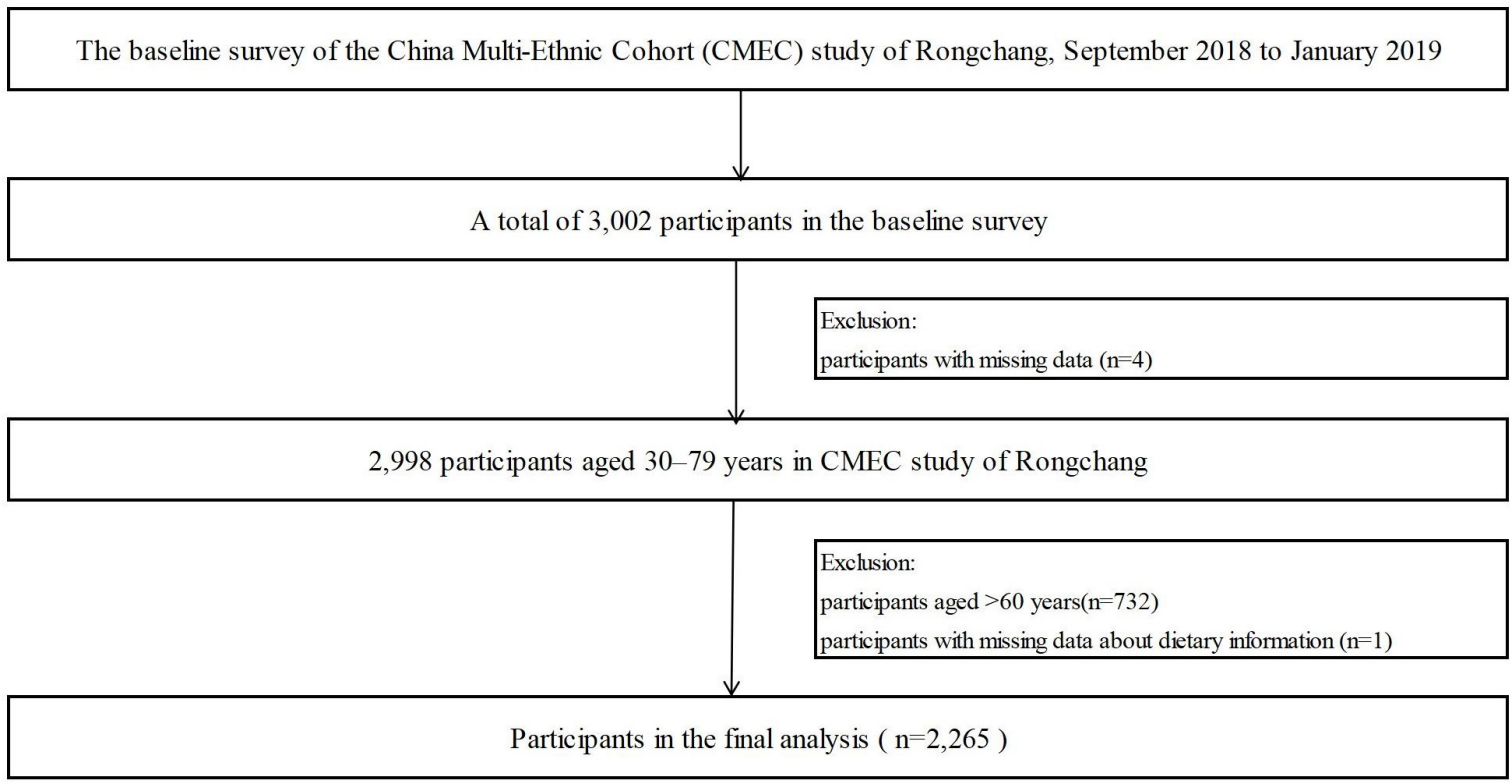
Flowchart illustrating the participant selection process at each stage.

Participants were (1) aged 30–60 years at the baseline survey in 2018, (2) had lived in Rongchang for at least 6 months, (3) of Han ethnicity, (4) voluntarily participating, willing to provide biological samples, and agreeing to undergo follow-up assessments; and (5) without history of mental illness, cognitive impairment, or communication barriers. We excluded participants who met any of the following criteria: (1) aged ≥60 years; (2) incomplete data on demographics, questionnaires, physical examination, or blood biochemistry. Initially, 3,002 participants were included in the baseline survey. After exclusion of four participants with missing data (These four participants only had their personal information registered in the system, but did not undergo questionnaire surveys, physical examinations, or other study procedures) and 732 participants aged ≥60 years, and one participant with missing data about dietary information, a total of 2,265 participants were retained for the final analysis (Figure 1).

Participants aged 61–79 years were excluded from the present study for the following reasons. First, previous studies had demonstrated that the prevalence of hyperuricemia was markedly elevated among older adults (21, 22). Second, elder adults frequently suffered from multiple chronic comorbidities. After adjustment for potential confounding factors, hyperuricemia remained significantly associated with hypertension and dyslipidemia (23), suggesting a complex interactive relationship between chronic diseases and hyperuricemia. Third, elder adults often have concomitant hypertension, dyslipidemia, diabetes, cardiovascular diseases, and other chronic conditions, and they routinely used medications such as diuretics, antihypertensive agents, and lipid-lowering drugs. These medications may interfere with uric acid metabolism, thereby confounding the true association between dietary factors (e.g., spicy food consumption) and hyperuricemia. Fourth, physiological renal function declined with advancing age, which may impair renal uric acid excretion (24). Most importantly, previous studies had indicated that both the frequency and intensity of spicy food consumption were positively associated with serum uric acid levels and the risk of hyperuricemia (25). However, this positive association was more pronounced among males and younger adults, whereas it was relatively weaker among females and elder adults. Based on these considerations, the age of participants included in this study was restricted to 30–60 years.

### 2.3 Data collection

The data collection process primarily involved questionnaire surveys, physical examinations, and laboratory tests. The questionnaire survey was administered using a specialized electronic questionnaire developed by the research team. Questionnaires and physical examinations were conducted face-to-face. (1) General Information: included name, age, gender, ethnicity, marital status, educational level, job status, average yearly income, smoking and daily alcohol consumption, personal and family history of disease, physical activity (PA) levels, sleep patterns, dietary habits, etc. (2) Physical examinations: mainly comprised measurements of height, weight, waist circumference (WC), blood pressure, and related indicators. (3) Laboratory tests: venous blood samples were collected from participants after at least 8 h of fasting, and were used to measure indicators such as blood glucose, total cholesterol (TC), triacylglycerol (TG), low density lipoprotein cholesterol (LDL-C), high-density lipoprotein cholesterol (HDL-C), uric acid (UA) and etc.

### 2.4 Methods of physical examination

Height and weight were measured using a standardized stadiometer and weighing device. Participants were instructed to remove shoes and hats, stand upright with the head, buttocks, and heels aligned against the stadiometer, and look straight ahead. The equipment had a maximum height capacity of 2.0 m and a maximum weight capacity of 150 kg. All instruments were verified by quality inspection authorities and calibrated prior to each use. Waist circumference (WC) was measured using a flexible tape, with the examiner positioned to the right of the participant and the tape placed horizontally around the abdomen at the midpoint between the lower border of the 12th rib and the iliac crest. Blood pressure was measured using an electronic sphygmomanometer. Three readings were taken at rest, with 1-min intervals between each measurement.

### 2.5 Assessment of covariates

#### 2.5.1 Basic characteristics

Age was expressed as Mean ± standard deviation (SD) and grouped into “30–39 years,” “40–49 years,” and “50–60 years.” Gender was grouped into “males” and “females.” Marital status was grouped into “married/cohabiting,” “separated/divorced/widowed/unmarried.” Education level was grouped into “primary school or below,” “junior middle school,” “high school or above.” Job status was grouped into “farmers,” “government employees,” “workers,” “sales & service staffs,” “others.” Average yearly income was grouped into “<20,000 yuan,” “20,000–59,999 yuan,” “60,000–99,999 yuan,” and “≥100,000 yuan.”

#### 2.5.2 Behavioral and lifestyle factors

Smoking status was grouped into “never,” “current.” Daily alcohol consumption was grouped into 0 g/d, < 10 g/d, 10∼19.99 g/d and ≥ 20 g/d. We investigated the drinking frequency, types of alcoholic beverages and alcohol content among participants in the past week, and calculated their daily alcohol consumption and all participants were divided into four groups. PA levels were assessed using metabolic equivalent of tasks (METs) values (26), quantifying PA across four domains: occupational, transportation-related, household, and leisure-time activities. Total weekly PA volume, expressed in MET-minutes per week (MET-min/wk), was categorized as follows: insufficient (< 600 MET-min/wk), moderate (600–3,000 MET-min/wk), or sufficient (> 3,000 MET-min/wk) (27). Sleep duration was categorized according to the sleep guidelines of national lung and blood institute recommendations, which define 7–8 hours per day as optimal for adults. Specifically, participants were classified into three groups (28): < 7 h/day (insufficient sleep), 7–8.9 h/day (sufficient sleep), and ≥ 9 h/day (excessive sleep).

#### 2.5.3 Dietary data

Dietary data were collected using a Food Frequency Questionnaire (FFQ) (29). In the baseline survey, Habitual consumption of 13 primary food groups was self-reported by all participants through the administered FFQ. Poultry and products, aquatic and seafood, wheat-based foods, fresh fruits, eggs and products, rice, legume products, red meat and products, fresh vegetables, pickled vegetables, potatoes and tubers, whole grains, dairy and products were included. For instance, eggs and products consumption were evaluated through a three-part question sequence. Participants first indicated whether they had consumed these items in the past year (Yes or No). Affirmative responders then reported their intake frequency (XX times per day/week/month/year) and typical serving size in grams. Based on the Chinese Food Composition Table 2004 (30), we calculated the average daily total intake for each participants.

#### 2.5.4 Medical status

Hypertension (31) was defined as an average systolic blood pressure (SBP) ≥140 mmHg and/or diastolic blood pressure (DBP) ≥90 mmHg, or a prior physician diagnosis of hypertension, or current use of antihypertensive medication or lifestyle modifications for blood pressure control. Diabetes mellitus (32) was diagnosed if the fasting plasma glucose (FPG) level was ≥ 7.00 mmol/L, or if the participant had a physician-confirmed diabetes, or was receiving active treatment for diabetes (e.g., glucose-lowering medication or prescribed lifestyle intervention). Dyslipidemia (33) was defined as the presence of at least one of the following lipid abnormalities: (1) TC ≥ 6.2 mmol/L; (2) TG ≥ 2.3 mmol/L; (3) LDL-C ≥ 4.1 mmol/L; or (4) HDL-C < 1.0 mmol/L. Hyperuricemia (34) was defined as a serum UA level ≥ 420 μmol/L. Body mass index (BMI) (35) was calculated as weight in kilograms divided by height in meters squared (kg/m^2^). In accordance with established criteria, participants were categorized as overweight if their BMI ranged from 24.0 to 27.9 kg/m^2^, and as obese if their BMI was ≥ 28.0 kg/m^2^ (36). Central obesity was defined as a waist circumference (WC) ≥ 90 cm in males and ≥ 85 cm in females (36).

#### 2.5.5 Definition of abnormal renal function

Estimated glomerular filtration rate (eGFR) was calculated for all participants using the creatinine-based Chronic Kidney Disease Epidemiology Collaboration (CKD-EPI) equation (37) and was expressed in mL/min/1.73 m^2^. According to the CKD staging criteria (38), participants were categorized into 6 stages. In this study, an eGFR < 90 mL/min/1.73 m^2^ was defined as abnormal renal function.

#### 2.5.6 Assessment of spicy food intake

The definition of spicy food intake covered all pungent flavorings applied in cooking or consumed at the table, including chili sauce, chili oil, fresh red peppers, dried chili peppers, or other hot spices (e.g., curry powder or similar spicy seasonings). Spicy food intake frequency was assessed using a five-point scale ranging from “never” to “6–7 days/week.” The “< 1 day/week” option received no responses. Among consumers reporting weekly intake, follow-up questions captured preferred spice types, including chili sauce, chili oil, fresh or dried peppers, and others, as well as self-reported flavor intensity. Trained interviewers asked participants the following standardized questionnaire item: “Do you usually prefer slightly spicy, moderately spicy, or extremely spicy food?” Participants were asked to select one response option.

#### 2.5.7 Assessment of numbing substances intake

Numbing substance intake referred to the use of any anesthetic-flavored condiments, whether incorporated during food preparation or added at the point of consumption, including Sichuan pepper oil, dried Sichuan pepper, fresh Sichuan pepper and others (e.g., pepper-containing spices). The assessment of numbing substances intake, including its frequency and flavor, followed the same methodology as that used for the spicy food intake survey.

For analytic clarity, participants were cross-classified into four mutually exclusive groups “both never,” “numbing only,” “spicy only,” “both numbing and spicy intake.” The assessments of numbing-spicy food intake were comparable to that adopted in the China Kadoorie Biobank (8). To quantify cumulative exposure, we constructed an exploratory numbing-spicy diet score by assigning ordinal values to each frequency category (never = 0, 1–2 days/week = 1, 3–5 days/week = 2, 6–7 days/week = 3) and summing the numbing and spicy components (range 0–6). Equal weight was assigned to both dimensions for simplicity and due to the lack of biological evidence supporting differential weighting. This approach represented an exploratory and straightforward scoring method. The cumulative frequency score was used as the evaluation indicator, with higher scores indicating a greater tendency to consume numbing and spicy foods concurrently.

### 2.6 Quality control

Prior to data collection, all study personnel, including interviewers and examining physicians, completed standardized training and qualification testing administered by the quality control team. The training covered the following key areas: (1) electronic devices (tablets or computers) for administering and recording questionnaire data; (2) procedures for verifying and uploading collected data; (3) standardized protocols for physical measurements; and (4) standardized handling workflows for biological sample collection, processing, and transportation. All questionnaires were reviewed and verified by designated quality control staff before being uploaded to the central database. In addition, trained postgraduate students from Sichuan University performed daily random checks on 1% of submitted questionnaires. Any identified discrepancies or issues were promptly communicated to the responsible investigator for clarification and correction. All questionnaire surveys and physical examinations were performed by physicians with the professional title of associate chief physician or above. Biochemical analyses were conducted independently by a certified external facility (Chongqing Dian Medical Testing Center Co., Ltd.).

## 3 Statistical analyses

Continuous variables were presented as mean ± standard deviation (SD) if they followed a normal distribution, as assessed by the Shapiro-Wilk test. Categorical variables were described by frequency counts and percentages [n (%)]. Differences in categorical characteristics between groups were compared by the chi-square (χ^2^) test.

### 3.1 Derivation and grouping of dietary patterns

Dietary patterns were derived using exploratory factor analysis. First, the suitability of the data for factor analysis was assessed by examining variable correlations via the Kaiser-Meyer-Olkin (KMO) measure and Bartlett’s test of sphericity (a KMO value > 0.6 and a *P* < 0.05 for Bartlett’s test were considered acceptable). The final number of factors to retain was determined based on the eigenvalue-greater-than-one criterion, inspection of the scree plot, and the interpretability of the factors. Varimax rotation was applied to achieve a clearer factor structure. Each extracted dietary pattern was named according to the food groups with the highest absolute factor loadings, typically the top one or two food groups. Based on the factor scores for each dietary pattern, participants were categorized into three groups (T1, T2, T3). T1 represented the lowest adherence (least consistent with the pattern), whereas T3 represented the highest adherence (most consistent with the pattern).

### 3.2 Association between numbing or/and spicy intake and hyperuricemia by logistic model

To examine the association between numbing or/and spicy intake and hyperuricemia, A stepwise covariate adjustment strategy (*P* < 0.05) was adopted in the multivariable logistic regression models. Covariates were sequentially adjusted for demographic characteristics, lifestyle factors, medical history, numbing–spicy dietary habits, and dietary patterns to evaluate their associations with hyperuricemia. All continuous independent variables included in the models were assessed for multicollinearity by variance inflation factor (VIF); a VIF < 5 was considered acceptable, confirming the absence of multicollinearity. A hierarchical modeling approach was employed for the regression analyses: Model 1: unadjusted crude model. Model 2: adjusted for gender, educational level, average yearly income. Model 3: Adjusted for smoking status, daily alcohol consumption, physical activity level based on Model 3. Model 4: adjusted for hypertension, dyslipidemia, overweight/obesity, central obesity based on Model 3. Model 5: based on Model 4, each of the extracted dietary patterns was individually introduced as an additional adjustment variable to independently assess its association with hyperuricemia. Categorical variables were coded using dummy variables, with appropriate reference categories specified. Subsequently, multivariable logistic regression was performed using backward elimination (likelihood ratio) to identify independent predictors of HUA. The full model initially included all candidate variables identified through univariate analysis (*P* < 0.05). Variables were sequentially removed if their exclusion did not significantly worsen the model fit (*P* > 0.10). This approach was preferred over forward selection because it preserved variables that may exhibit suppressor effects or confounding patterns only apparent in the presence of other covariates (α_*in*_ = 0.05, α_*out*_ = 0.10). Given that only 156 participants reported consuming neither numbing nor spicy foods, whereas 1,537 participants consumed both, the use of such a small reference group may lead to unstable association estimates, particularly in fully adjusted models with multiple covariates. Therefore, the reference groups were redefined by using participants who consumed only spicy foods (Model 11), only numbing foods (Model 12), or either numbing or spicy foods (Model 13) as separate control groups. Subsequently, the association between combined numbing and spicy food consumption and hyperuricemia was further evaluated.

### 3.3 The association between numbing or/and spicy intake and hyperuricemia by restricted cubic spline

To visually explore the functional form of the association, a “numbing and/or spicy frequency score” was constructed by assigning points according to the frequency of numbing and/or spicy food consumption. Restricted cubic spline (RCS) analysis was subsequently applied to examine the association between the composite numbing–spicy frequency score (range: 0–6; treated as a continuous variable) and the risk of hyperuricemia using logistic regression models with four knots. We acknowledge that this score represents an ordinal composite measure rather than a true continuous biological exposure metric. Therefore, the RCS findings should be interpreted with caution and complemented by categorical trend analyses. The overall association and potential nonlinearity were evaluated using the *P*_ for overall_ and *P*_ for_ _*nonlinear*_ tests, respectively. A significant (*P*_ for overall_ < 0.05) indicates an association between numbing or/and spicy intake and hyperuricemia, while a significant *P*_ for_ _*nonlinear*_ (*P*_ for_ _*nonlinear*_ < 0.05) suggests that the association deviates from linearity, implying a nonlinear dose-response relationship.

### 3.4 Interaction analysis between numbing or/and spicy intake and hyperuricemia

To further investigate the combined associations of numbing and/or spicy intake on the risk of hyperuricemia, analyses were conducted from both multiplicative and additive interaction perspectives. (1) Multiplicative interaction: a product term between the categorized numbing and/or spicy intake variables was included in a logistic regression model along with the main effects. The significance of this product term was assessed through likelihood ratio test to evaluate the presence of multiplicative interaction. (2) Additive interaction: to assess the joint effect on an additive scale, measures of additive interaction were calculated, including the relative excess risk due to interaction (RERI), the attributable proportion due to interaction (AP), and the synergy index (SI). These indices quantify whether the observed risk when both factors are present exceeds the sum of the risks associated with each factor alone.

### 3.5 Exploratory mediation analysis between numbing or/and spicy intake and hyperuricemia mediated by dietary pattern

**FIGURE 2:**
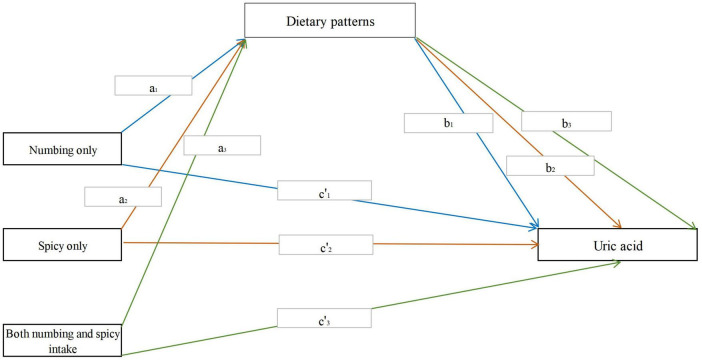
Exploratory mediation analysis for multicategorical independent variable (participants whom never consumed numbing nor spicy food grouped “Both never” were chosen for reference group).

To investigate whether dietary patterns mediate the association between types of numbing or/and spicy intake and serum uric acid levels, an exploratory mediation analysis was conducted. The variables were specified as follows: the independent variable (X) was the type of numbing or/and spicy intake, treated as a four category variables (“both never,” “spicy only,” “numbing only,” “both numbing and spicy”). Using the “both never” group as the reference, three dummy variables were created for the analysis. The mediator (M) was the dietary pattern, represented by its continuous factor scores. The outcome variable (Y) was the continuous serum uric acid level. Mediation analysis for multicategorical independent variables followed the approach proposed by Hayes and Preacher (39). The mediation association was tested using a bootstrap approach based on ordinary least squares regression. The percentile bootstrap method with 5,000 resamples was applied to estimate the mediation association and its 95%CI. A mediation association was considered statistically significant if the 95%CI did not include zero (40). The mediation association diagram for the multicategorical independent variable was illustrated in Figure 2. Given the cross-sectional design of the study, the temporal sequence among exposure, mediator, and outcome variables could not be determined. Therefore, the mediation analyses were considered strictly exploratory and descriptive, and the term “mediation” was used to describe the statistical partitioning of covariance rather than to imply evidence of a causal biological pathway.

### 3.6 Sensitivity and subgroup analyses

To ensure the robustness of the findings, the following supplementary analyses were performed:

(1) sensitivity analysis: the primary multivariable logistic regression analysis was repeated after excluding participants with abnormal renal function at baseline (defined as eGFR < 90 mL/min/1.73 m^2^) and excluding 925 participants with hypertension, diabetes, dyslipidemia; (2) subgroup analysis: participants were stratified by key characteristics such as age, sex, BMI, dietary patterns and other factors. The primary analysis was then repeated within each subgroup to examine the consistency of the association across different populations. All statistical analyses were employed by SPSS (version 27.0) and R (version 4.3.2) software. A two-sided *P* < 0.05 was considered statistically significant.

## 4 Results

### 4.1 Basic characteristics of the participants by hyperuricemia

The basic characteristics of the participants by hyperuricemia were presented in Table 1. A total of 2,265 participants were included in the analyses, with a mean age of 45.21 years (SD = 7.84). The median (interquartile range) serum uric acid concentration was 319 (263,377) μmol/L, and the overall prevalence of hyperuricemia was 13.20% (299/2,265). Significant variations in hyperuricemia prevalence were observed among categories of gender, educational level, average yearly income, smoking status, daily alcohol consumption, PA level, sleep duration, hypertension, dyslipidemia, overweight/obesity, central obesity (all *P* < 0.05).

**TABLE 1:** Basic characteristics of the participants by hyperuricemia.

Variables	Total	Hyperuricemia		Statistic	*P*
	(*n* = 2,265)	No (*n* = 1,966)	Yes (*n* = 299)		
Age, years				1.247	0.264
30–39	594 (26.22)	498 (83.84)	96 (16.16)		
40–49	990 (43.70)	881 (89.99)	109 (11.01)		
50–60	682 (30.11)	681 (86.20)	94 (13.80)		
Gender				166.351	< 0.001
Males	1,122 (49.54)	870 (77.54)	252 (22.46)		
Females	1,143 (50.46)	1,096 (95.89)	47 (4.11)		
Marital status				0.402	0.526
Married/cohabiting	2,093 (92.41)	1,814 (86.67)	279 (13.33)		
Separated/divorced/widowed/ unmarried	172 (7.59)	152 (88.37)	20 (11.63)		
Educational level				7.406	0.025
Primary school or below	528 (23.31)	444 (84.09)	84 (15.91)		
Junior middle school	510 (22.52)	458 (89.80)	52 (10.20)		
High school or above	1,227 (54.17)	1,064 (86.72)	163 (13.28)		
Job status				5.510	0.239
Farmers	660 (29.14)	578 (87.58)	82 (12.42)		
Government employee	304 (13.42)	256 (84.21)	48 (15.79)		
Worker	259 (11.43)	225 (86.87)	34 (13.13)		
Sales and service staff	502 (22.16)	427 (85.06)	75 (14.94)		
Others	540 (23.84)	480 (88.89)	60 (11.11)		
Average yearly income, yuan				20.109	< 0.001
<20,000	622 (27.46)	564 (90.68)	58 (9.32)		
20,000–59,999	823 (36.34)	720 (87.48)	103 (12.52)		
60,000–99,999	417 (18.41)	354 (84.89)	63 (15.11)		
≥ 100,000	403 (17.79)	328 (81.39)	75 (18.61)		
Smoking status				46.334	< 0.001
Never	1,810 (79.91)	1,615 (89.23)	195 (10.77)		
Current	455 (20.09)	351 (77.14)	104 (22.86)		
Daily alcohol consumption, g/d				23.435	< 0.001
0	1,072 (47.28)	962 (89.82)	109 (10.18)		
<10	649 (28.65)	566 (87.21)	83 (12.79)		
10–19.99	186 (8.21)	152 (81.72)	34 (18.28)		
≥ 20	359 (15.85)	290 (80.78)	69 (19.22)		
PA level				6.753	0.034
Insufficient	230 (10.16)	204 (88.70)	26 (11.30)		
Moderate	257 (11.36)	235 (91.44)	22 (8.56)		
Sufficient	1,776 (78.48)	1,526 (85.92)	250 (14.08)		
Sleep duration				2.917	0.088
Insufficient	743 (32.80)	632 (85.06)	111 (14.94)		
Sufficient	1,522 (67.20)	1,334 (87.65)	188 (12.35)		
Hypertension				10.782	0.001
No	1,571 (69.36)	1,388 (88.35)	183 (11.65)		
Yes	694 (30.64)	578 (83.29)	116 (16.71)		
Diabetes				0.064	0.800
No	2,084 (92.01)	1,810 (86.85)	274 (13.15)		
Yes	181 (7.99)	156 (86.19)	25 (13.81)		
Dyslipidemia				107.472	< 0.001
No	1,635 (72.19)	1,494 (91.38)	141 (8.62)		
Yes	630 (27.81)	472 (74.92)	158 (25.08)		
Overweight/obesity				36.185	< 0.001
No	993 (43.84)	910 (91.64)	83 (8.36)		
Yes	1,272 (56.16)	1,056 (83.02)	216 (16.98)		
Central obesity				35.544	< 0.001
No	1,720 (75.94)	1,534 (89.19)	186 (10.81)		
Yes	545 (24.06)	432 (79.27)	113 (20.73)		

Analysis by χ^2^-test. PA, physical activity.

### 4.2 Numbing and spicy characteristics of the participants by hyperuricemia

**FIGURE 3:**
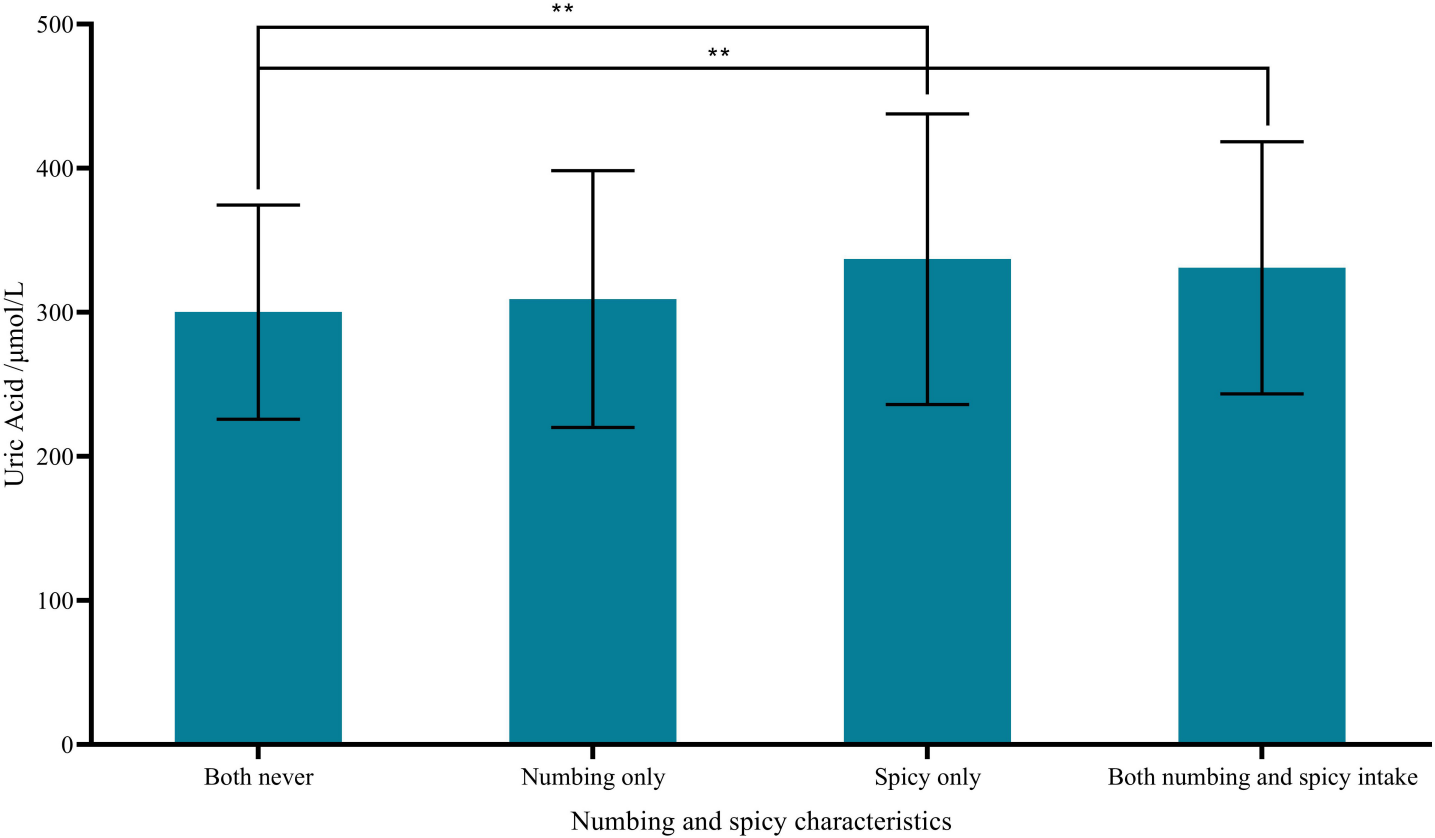
Serum uric acid levels across different numbing and spicy intake groups. **P < 0.05.

The numbing and spicy characteristics of the participants by hyperuricemia were presented in Table 2. Participants who consumed numbing substances had a significantly higher prevalence of hyperuricemia than non-consumers; the prevalence rose progressively with increasing frequency and intensity of numbing flavor (all *P* < 0.05). The intake of Sichuan pepper oil showed the highest prevalence of hyperuricemia (*P* < 0.05). Similar associations were observed for spicy food: consumers of spicy intake, those who ate them more frequently, those who preferred stronger flavor, and intake of chili sauce or fresh chili pepper all demonstrated higher prevalences of hyperuricemia (all *P* < 0.05). Those whom regularly consumed both numbing and spicy foods showed the highest prevalence of hyperuricemia (15.61%), which was significantly greater than the prevalence observed in participants who consumed only numbing, only spicy, or neither type of substances (*P* < 0.05). Consistent with these findings, serum uric acid concentrations differed significantly across the four consumption groups (“both never,” “numbing only,” “spicy only,” “both numbing and spicy”; Kruskal–Wallis *H* = 26.75, *P* < 0.001). Pairwise comparisons showed that both the “spicy-only” group (*P* = 0.005) and the “both numbing and spicy intake” group (*P* <0.001) had higher UA levels than participants who consumed “both never” (Figure 3).

**TABLE 2:** Numbing and spicy characteristics of the participants by hyperuricemia.

Variables	Total	Hyperuricemia		Statistic	*P*
	(*n* = 2,265)	No (*n* = 1,966)	Yes (*n* = 299)		
Numbing substances intake				15.094	< 0.001
No	473 (20.88)	436 (92.18)	37 (7.82)		
Yes	1,792 (79.12)	1,530 (85.38)	262 (14.62)		
Numbing frequency				16.795	0.002
Never	473 (20.88)	436 (92.18)	37 (7.82)		
1–2 d/week	567 (25.03)	487 (85.89)	80 (14.11)		
3–5 d/week	193 (8.52)	162 (8.52)	31 (16.06)		
6–7 d/week	1,032 (45.46)	881 (85.37)	151 (14.63)		
Numbing flavor				19.615	< 0.001
Never	473 (20.88)	436 (92.18)	37 (7.82)		
Slightly numbing	1,591 (70.24)	1,368 (70.24)	223 (14.02)		
Moderately numbing	180 (7.95)	145 (7.95)	35 (19.44)		
Extremely numbing	21 (0.93)	17 (0.93)	4 (19.05)		
Numbing substances				24.076	< 0.001
Never	473 (20.88)	436 (92.10)	37 (7.82)		
Sichuan pepper oil	182 (8.04)	147 (80.77)	35 (19.23)		
Dried Sichuan pepper	1,115 (49.23)	959 (86.01)	156 (13.99)		
Fresh Sichuan pepper	234 (10.33)	209 (89.32)	25 (10.68)		
Other (pepper-containing spices)	261 (11.52)	215 (82.38)	46 (17.62)		
Spicy intake				17.737	< 0.001
No	410 (18.10)	382 (93.17)	28 (6.83)		
Yes	1,855 (81.90)	1,584 (85.39)	271 (14.61)		
Spicy frequency				25.634	< 0.001
Never	410 (18.10)	382 (93.17)	28 (6.83)		
1–2 d/week	351 (15.50)	308 (87.75)	43 (12.25)		
3–5 d/week	217 (9.58)	173 (79.72)	44 (20.28)		
6–7 d/week	1,287 (56.82)	1,103 (85.70)	184 (14.30)		
Spicy flavor				18.933	< 0.001
Never	410 (18.10)	382 (93.17)	28 (6.83)		
Slightly spicy	1,551 (68.48)	1,330 (85.75)	221 (14.25)		
Moderately spicy	238 (10.51)	198 (83.19)	40 (16.81)		
Extremely spicy	66 (2.91)	56 (84.85)	10 (15.15)		
Chili sauce				16.038	< 0.001
No	444 (19.60)	411 (92.57)	33 (7.43)		
Yes	1,821 (80.40)	1,555 (85.39)	266 (14.61)		
Chili oil				2.610	0.106
No	1,421 (62.74)	1,246 (87.68)	175 (12.32)		
Yes	844 (37.26)	720 (85.31)	124 (14.69)		
Dried chili pepper				2.272	0.132
No	1,288 (56.87)	1,130 (87.73)	158 (12.27)		
Yes	977 (43.13)	836 (85.57)	141 (14.43)		
Fresh chili pepper				17.918	< 0.001
No	538 (23.75)	496 (92.19)	42 (7.81)		
Yes	1,727 (76.25)	1,470 (85.12)	257 (14.88)		
Other hot spices				0.006	0.937
No	2,018 (89.09)	1,752 (86.82)	266 (13.18)		
Yes	247 (10.91)	214 (86.64)	33 (13.36)		
Numbing and/or spicy intake				27.625	< 0.001
Both never	156 (6.89)	150 (96.15)	6 (3.85)		
Numbing only	255 (11.26)	233 (91.37)	22 (8.63)		
Spicy only	317 (14.00)	286 (90.22)	31 (9.78)		
Both numbing and spicy intake	1,537 (67.86)	1,297 (84.39)	240 (15.61)		

Analysis by χ^2^-test.

### 4.3 Dietary patterns of the participants by hyperuricemia

**FIGURE 4:**
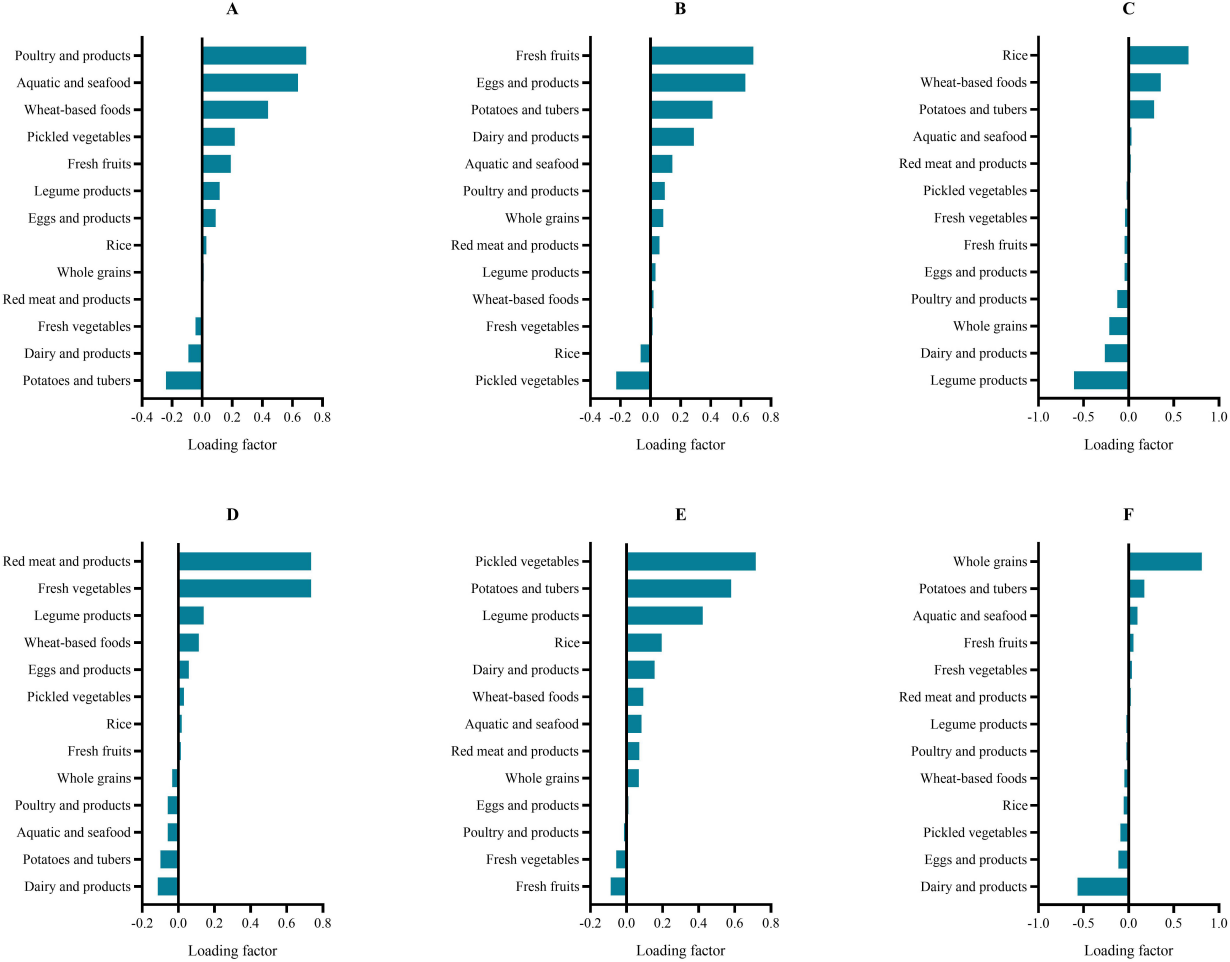
Six dietary patterns identified through factor analysis. **(A)** Poultry-aquatic dietary pattern; **(B)** fruit-egg dietary pattern; **(C)** rice-soy product dietary pattern; **(D)** red meat-vegetable dietary pattern; **(E)** pickled vegetable-Potato dietary pattern; **(F)** whole grain dietary pattern.

Prior to conducting factor analysis, we calculated the Kaiser-Meyer-Olkin (KMO) measure and performed Bartlett’s test of sphericity. The results indicated that factor analysis was appropriate (KMO = 0.654; Bartlett’s test: χ^2^ = 492.081, *P* < 0.001). Six dietary patterns were identified through factor analysis and collectively explained 53.40% of the total variance (with the scree plot shown in Supplementary Figure 1. The factor loadings for each dietary pattern were presented in Figure 4 (Supplementary Table 1). Each dietary pattern was labeled according to the one or two items with the highest loadings, including (1) poultry-aquatic dietary pattern, (2) fruit-egg dietary pattern, (3) rice-soy product dietary pattern, (4) red meat-vegetable dietary pattern, (5) pickled vegetable-potato dietary pattern and (6) whole grain dietary pattern. Participants were assigned to tertiles (T1–T3) based on factor scores, with higher scores indicating closer adherence. The dietary patterns of the participants by hyperuricemia were presented in Table 3. The highest prevalence of hyperuricemia was observed in the T3 groups of the poultry–aquatic and rice–soy product dietary patterns. In contrast, the T3 group of the fruit–egg dietary pattern showed the lowest prevalence of hyperuricemia (all *P* < 0.05). However, no statistically significant differences in hyperuricemia prevalence were observed across tertiles of the red meat–vegetable, pickled vegetable–potato, or whole grain dietary patterns (all *P* > 0.05).

**TABLE 3:** Prevalence of hyperuricemia across different dietary patterns.

Variables	Total	Hyperuricemia		Statistic	*P*
	(*n* = 2,265)	No (*n* = 1,966)	Yes (*n* = 299)		
Poultry-aquatic dietary pattern				14.575	< 0.001
T1	756 (33.38)	679 (89.81)	77 (10.19)		
T2	758 (33.47)	662 (87.34)	96 (12.66)		
T3	751 (33.16)	625 (83.22)	126 (16.78)		
Fruit-egg dietary pattern				13.289	< 0.001
T1	768 (33.91)	639 (83.20)	129 (16.80)		
T2	742 (32.76)	655 (88.27)	87 (11.73)		
T3	755 (33.33)	672 (89.01)	83 (10.99)		
Rice-soy product dietary pattern				8.513	0.014
T1	754 (33.29)	661 (87.67)	93 (12.33)		
T2	758 (33.47)	673 (88.79)	85 (11.21)		
T3	753 (33.25)	632 (83.93)	121 (16.07)		
Red meat-vegetable dietary pattern				0.202	0.904
T1	760 (33.55)	662 (87.11)	98 (12.89)		
T2	750 (33.11)	652 (86.93)	98 (13.07)		
T3	755 (33.33)	652 (86.36)	103 (13.64)		
Pickled vegetable-potato dietary pattern				1.767	0.413
T1	750 (33.11)	661 (88.13)	89 (11.87)		
T2	765 (33.77)	660 (86.27)	105 (13.73)		
T3	750 (33.11)	645 (86.00)	105 (14.00)		
Whole grain dietary pattern				0.619	0.734
T1	751 (33.16)	656 (87.35)	95 (12.65)		
T2	759 (33.51)	653 (86.03)	106 (13.97)		
T3	755 (33.33)	657 (87.02)	98 (12.98)		

Analysis by χ^2^-test.

### 4.4 Basic characteristics of the participants by numbing or/and spicy intake

The basic characteristics of the participants by numbing or/and spicy intake were presented in Table 4. Of the 2,265 participants, 156 reported never consuming either numbing or spicy foods, whereas 1,537 (67.86%) consumed both numbing and spicy food. Across the four consumption groups, significant differences were observed for age, gender, educational level, average yearly income, smoking status, daily alcohol consumption, sleep duration, hypertension, dyslipidemia, hyperuricemia, overweight/obesity and central obesity (all *P* < 0.05). Participants who consumed both numbing and spicy foods exhibited the highest prevalence of most characteristics (age, gender, average yearly income, smoking status, daily alcohol consumption, sleep duration, hypertension, dyslipidemia, hyperuricemia, overweight/obesity and central obesity). By contrast, the “spicy-only” group contained the largest proportion in different subgroup of educational level.

**TABLE 4:** Basic characteristics of the participants by numbing or/and spicy intake.

Variables	Total (*n* = 2,265)	Both never (*n* = 156)	Spicy only (*n* = 317)	Numbing only (*n* = 255)	Both numbing and spicy intake (*n* = 1,537)	Statistic	*P*
Age/years						15.536	0.016
30–39	594 (26.22)	41(6.90)	79 (13.30)	61 (10.27)	413 (69.53)		
40–49	990 (43.70)	84(8.48)	140 (14.14)	126 (12.73)	640 (64.65)		
50–60	682 (30.11)	31(4.55)	98 (14.39)	68 (9.99)	484 (71.07)		
Gender						13.059	0.005
Males	1,122 (49.54)	68 (6.06)	164 (14.62)	103 (9.18)	787 (70.14)		
Females	1,143 (50.46)	88 (7.70)	153 (13.39)	152 (13.30)	750 (65.62)		
Marital status						2.345	0.504
Married/cohabiting	2,093 (92.41)	146 (6.98)	291 (13.90)	241 (11.51)	1,415 (67.61)		
Separated/divorced/ widowed/unmarried	172 (7.59)	10 (5.81)	26 (15.12)	14 (8.14)	122 (70.93)		
Educational level						13.525	0.035
Primary school or below	528 (23.31)	32 (6.06)	379 (71.78)	54 (10.23)	63 (11.93)		
Junior middle school	510 (22.52)	38 (7.45)	330 (64.71)	50 (9.80)	92 (18.04)		
High school or above	1,227 (54.17)	86 (7.01)	828 (67.48)	151 (12.31)	162 (13.20)		
Job status						17.548	0.13
Farmers	660 (29.14)	45 (6.82)	102 (15.45)	70 (10.61)	443 (67.12)		
Government employee	304 (13.42)	22 (7.24)	37 (12.17)	31 (10.20)	214 (70.39)		
worker	259 (11.43)	20 (7.72)	37 (14.29)	26 (10.04)	176 (67.95)		
Sales and service staff	502 (22.16)	32 (6.37)	67 (13.35)	44 (8.76)	359 (71.51)		
Others	540 (23.84)	37 (6.85)	74 (13.70)	84 (15.56)	345 (63.89)		
Average yearly income, yuan						39.659	< 0.001
<20,000	622 (27.46)	62 (9.97)	106 (17.04)	82 (13.18)	372 (59.81)		
20,000–59,999	823 (36.34)	46 (5.59)	119 (14.46)	95 (11.54)	563 (68.41)		
60,000–99,999	417 (18.41)	31 (7.43)	48 (11.51)	44 (10.55)	294 (70.50)		
≥ 100,000	403 (17.79)	17 (4.22)	44 (10.92)	34 (8.44)	308 (76.43)		
Smoking status						37.586	< 0.001
Never	1,810 (79.91)	144 (7.96)	258 (14.25)	227 (12.54)	1,181 (65.25)		
Current	455 (20.09)	12 (2.64)	59 (12.97)	28 (6.15)	356 (78.24)		
Daily alcohol consumption, g/d						93.181	< 0.001
0	1,072 (47.28)	121 (11.29)	205 (19.12)	122 (11.38)	624 (58.21)		
<10	649 (28.65)	21 (3.24)	62 (9.55)	77 (11.86)	489 (75.35)		
10–19.99	186(8.21)	7 (3.76)	21 (11.29)	21 (11.29)	137 (73.66)		
≥20	359 (15.85)	7 (1.95)	29 (8.08)	35 (9.75)	288 (80.22)		
PA level						6.27	0.394
Insufficient	230 (10.16)	17 (7.39)	26 (11.30)	42 (18.26)	145 (63.04)		
Moderate	257 (11.36)	22 (8.56)	32 (12.45)	33 (12.84)	170 (66.15)		
Sufficient	1,776 (78.48)	117 (6.59)	196 (11.04)	242 (13.63)	1,221 (68.75)		
Sleep duration						9.676	0.022
Insufficient	743 (32.80)	36 (4.85)	100 (13.46)	77 (10.36)	530 (71.33)		
Sufficient	1,522 (67.20)	120 (7.88)	217 (14.26)	178 (11.70)	1,007 (66.16)		
Hypertension						18.438	< 0.001
No	1,571 (69.36)	123 (7.83)	194 (12.35)	187 (11.90)	1,067 (67.92)		
Yes	694 (30.64)	33 (4.76)	123 (17.72)	68 (9.80)	470 (67.72)		
Diabetes						5.044	0.169
No	2,084 (92.01)	150 (7.20)	286 (13.72)	235 (11.28)	1,413 (67.80)		
Yes	181 (7.99)	6 (3.31)	31 (17.13)	20 (11.05)	124 (68.51)		
Dyslipidemia						10.609	0.014
No	1,635 (72.19)	124 (7.58)	214 (13.09)	196 (11.99)	1,101 (67.34)		
Yes	630 (27.81)	32 (5.08)	103 (16.35)	59 (9.37)	436 (69.21)		
Hyperuricemia						27.625	< 0.001
No	1,966 (86.80)	150 (7.63)	286 (14.55)	233 (11.85)	1,297 (65.97)		
Yes	299 (13.20)	6 (2.01)	31 (10.37)	22 (7.36)	240 (80.27)		
Overweight/obesity						222.118	< 0.001
No	993 (43.84)	140 (14.10)	73 (7.35)	153 (15.41)	627 (63.14)		
Yes	1,272 (56.16)	16 (1.26)	244 (19.18)	102 (8.02)	910 (71.54)		
Central obesity						28.228	< 0.001
No	1,720 (75.94)	140 (8.14)	235 (13.66)	212 (12.33)	1,133 (65.87)		
Yes	545 (24.06)	16 (2.94)	82 (15.05)	43 (7.89)	404 (74.13)		

Analysis by χ^2^-test.

### 4.5 The associations between numbing or/and spicy intake and hyperuricemia

**FIGURE 5:**
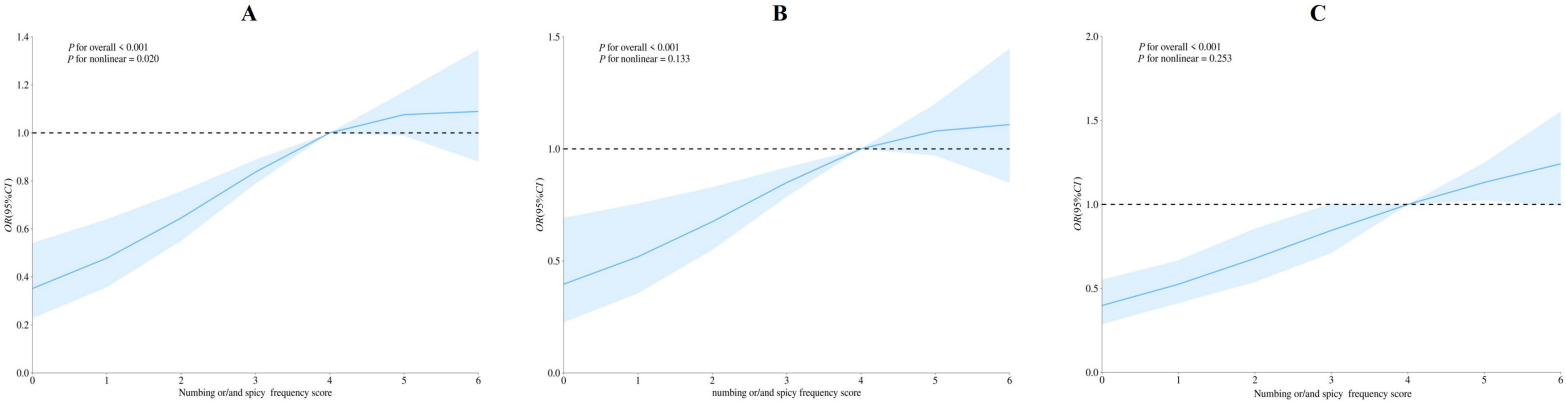
Association between numbing and/or spicy frequency score and hyperuricemia by restricted cubic splines. **(A)** Adjusted for gender, educational level, average yearly income, smoking status, daily alcohol consumption, physical activity level, hypertension, dyslipidemia, overweight/obesity, central obesity and poultry-aquatic dietary pattern; **(B)** sensitivity analysis after excluding 381 participants with renal dysfunction, the adjusted factors as **(A)**; **(C)** sensitivity analysis after excluding 925 participants with hypertension, diabetes, dyslipidemia, the adjusted factors as **(A)**. OR, odds ratio; CI, confidence interval.

The associations between numbing or/and spicy intake and hyperuricemia were presented in Table 5. No multicollinearity was detected among variables in the logistic regression models (Supplementary Table 2). In crude model without adjusting for any factors (Supplementary Table 3), The ORs in participants with numbing only, spicy only and both numbing and spicy intake were 2.361 (95%CI: 0.935–5.957, *P* = 0.069), 2.710 (95%CI: 1.106–6.640, *P* = 0.029), 4.626 (95%CI: 2.022–10.583, *P* < 0.001), respectively, versus the participants who neither consumed numbing nor spicy food. After successive adjustment, including age, educational level, average yearly income, smoking status, daily alcohol consumption, physical activity level, hypertension, dyslipidemia, overweight/obesity, central obesity, the Model 4 indicated that spicy only, both numbing and spicy intake were positively associated with hyperuricemia, with the ORs were 3.354 (95%CI: 1.610–6.987, *P* = 0.001), 2.628 (95%CI: 1.320–5.230, *P* = 0.006), respectively (Supplementary Table 6). However, numbing only intake was no longer significantly associated with hyperuricemia, with OR of 1.934 (95%CI: 0.885–4.229, *P* = 0.098). Additional adjustment for the six identified dietary patterns (Supplementary Tables 7–12), the similar association was only found in poultry-aquatic dietary pattern, with the ORs of 1.526 (95%CI: 1.009–2.308, *P* = 0.045) in spicy only and 2.689 (95%CI: 2.045–3.536, *P* < 0.001) in both numbing and numbing intake. However, no association was found in spicy only intake (*OR* = 1.806, 95%CI: 0.774–4.214, *P* = 0.171). In addition, when taking participants with only spicy food intake, only numbing food intake, and either numbing or spicy food intake as the reference groups, respectively, we similarly found that both numbing and spicy intake were positively associated with hyperuricemia (Supplementary Table 13). A composite “numbing-spicy score” was created from the frequency of combined intake. Restricted cubic spline, adjusted for the same confounding factors, showed a nonlinear, positive association with hyperuricemia (*P*_*for overall*_ < 0.001, *P*_ for_ _*nonlinear*_ = 0.020) (Figure 5A).

**TABLE 5:** The association between numbing or/and spicy intake and hyperuricemia by logistic regression analysis [OR(95%CI)].

Variables	Both never	Numbing only	Spicy only	Both numbing and spicy intake	P for trend
Model 1	1.000 (reference)	2.361 (0.935–5.957)^#^	2.710 (1.106–6.640)*	4.626 (2.022–10.583)^***^	0.029
Model 2	1.000 (reference)	3.157 (1.482–6.722)^**^	2.168 (0.968–4.858)^#^	2.432 (1.201–4.926)*	0.527
Model 3	1.000 (reference)	3.040 (1.426–6.482)^**^	2.073 (0.923–4.653)^#^	2.230 (1.097–4.531)*	0.592
Model 4	1.000 (reference)	3.354 (1.610–6.987)^**^	1.934 (0.885–4.229)^#^	2.628 (1.320–5.230)^**^	0.554
Model 5	1.000 (reference)	1.526 (1.009–2.308)*	1.806 (0.774–4.214)^#^	2.689 (2.045–3.536)^***^	0.018
Model 6	1.000 (reference)	2.209 (0.980–4.976)^#^	1.889 (0.807–4.422)^#^	1.566 (1.034–2.373)^#^	0.719
Model 7	1.000 (reference)	2.056 (0.918–4.606)^#^	1.779 (0.764–4.138)^#^	1.694 (0.799–3.592)^#^	0.538
Model 8	1.000 (reference)	2.051 (0.914–4.602)^#^	1.768 (0.758–4.122)^#^	1.701 (0.802–3.609)^#^	0.452
Model 9	1.000 (reference)	2.093 (0.933–4.698)^#^	1.868 (0.800–4.363)^#^	1.775 (0.835–3.774)^#^	0.436
Model 10	1.000 (reference)	2.090 (0.931–4.689)^#^	1.797 (0.771–4.188)^#^	1.709 (0.806–3.627)^#^	0.452

^#^*P* > 0.05; *P < 0.05; **P < 0.01; ***P < 0.001. OR, odds ratio; CI, confidence interval. Model 1: unadjusted. Model 2: Adjusted for gender, educational level, average yearly income; Model 3: Adjusted for smoking status, daily alcohol consumption, physical activity level based on Model 2; Model 4: Adjusted for hypertension, dyslipidemia, overweight/obesity, central obesity based on Model 3; Model 5: Model 4 plus poultry-aquatic dietary pattern; Model 6: Model 4 plus fruit-egg dietary pattern; Model 7: Model 4 plus rice-soy product dietary pattern; Model 8: Model 4 plus red meat-vegetable dietary pattern; Model 9: Model 4 plus pickled vegetable-Potato dietary pattern; Model 10: Model 4 plus whole grain dietary pattern.

### 4.6 Sensitivity analyses for the associations between numbing or/and spicy intake and hyperuricemia

After excluding 381 participants with renal dysfunction, 1,884 participants were included in the sensitivity analysis. After adjusting for confounding factors, logistic regression analysis showed that, compared to those who consumed neither numbing nor spicy food, the ORs for spicy only, numbing only and both numbing and spicy intake were 3.038 (95%CI: 1.238–7.456, *P* = 0.015), 1.552 (95%CI: 0.581–4.141, *P* = 0.380), 2.674 (95%CI: 1.158–6.175, *P* = 0.021) (Supplementary Table 14). Similar associations were still observed after excluding 925 participants with hypertension, diabetes, and dyslipidemia (Supplementary Table 15). Thus, the positive association was robust only for combined numbing and spicy consumption. RCS also demonstrated the similar association between “numbing-spicy score” and hyperuricemia after excluding participants with renal dysfunction (*P*_*for overall*_ < 0.001) (Figure 5B) or with hypertension, diabetes, and dyslipidemia (Figure 5C). However, nonlinear association was not observed (*P*_*for*_ _*nonlinear*_ = 0.133, *P*_*for*_ _*nonlinear*_ = 0.253), indicating a monotonic increase in the risk of hyperuricemia with higher “numbing-spicy score” in general population. Notably, we observed no evidence of a threshold effect. RCS analysis indicated that the association between numbing-spicy score and hyperuricemia presented a non-linear association among participants with renal dysfunction (Supplementary Figure 2A) or participants with hypertension, diabetes and dyslipidemia (Supplementary Figure 2B). Subgroup analyses across demographic, behavioral, metabolic, and dietary-pattern strata for the association between numbing or/and spicy intake and hyperuricemia by logistic regression analysis were conducted. In several subgroups, the association between combined numbing-spicy food intake and hyperuricemia remained detectable (Supplementary Table 16).

### 4.7 Interactions between numbing and/or spicy intake and hyperuricemia

The multiplicative interactions between numbing and/or spicy food intake and hyperuricemia are presented in Table 6. After adjusting for confounding factors, no significant multiplicative interactions were observed in the crude, adjusted, or sensitivity model. Regarding additive interactions, after adjustment for confounding factors, combined numbing and spicy food intake was positively associated with hyperuricemia, with an OR of 3.417 (95% CI: 1.419–8.227, *P* = 0.006) compared with the absence of both exposures. However, formal additive interaction indices, including the relative excess risk due to interaction (RERI), attributable proportion (AP), and synergy index (SI), were not statistically significant (Table 7), suggesting that the observed relationship reflected a joint association rather than evidence of biological synergy. Sensitivity analyses further demonstrated a consistent association between numbing–spicy food consumption and hyperuricemia. A bar chart illustrating the additive interaction association is provided in Supplementary Figure 3.

**TABLE 6:** Multiplicative interaction of numbing and spicy intake on hyperuricemia by logistic regression model.

Variables	β	S.E	Wals χ^2^	*OR*	95%CI	*P*-value
Model 1						
Intercept	−3.219	0.416	−7.734	0.040	0.018–0.090	<0.001
Numbing	0.997	0.457	2.181	2.710	1.106–6.638	0.029
Spicy	0.863	0.472	1.828	2.371	0.940–5.982	0.068
Numbing × spicy	−0.329	0.513	−0.641	0.720	0.263–1.969	0.522
Model 2						
Intercept	−2.449	0.547	−4.481	0.086	0.030–0.252	<0.001
Numbing	0.858	0.497	1.724	2.358	0.889–6.251	0.085
Spicy	0.518	0.487	1.065	1.679	0.647–4.361	0.287
Numbing × spicy	−0.147	0.543	−0.272	0.863	0.298–2.502	0.786
Model 3						
Intercept	−3.504	0.507	−6.905	0.030	0.011–0.801	<0.001
Numbing	0.955	0.561	1.704	2.600	0.866–7.801	0.088
Spicy	0.609	0.595	1.024	1.838	0.573–5.894	0.306
Numbing × spicy	−0.016	0.646	−0.025	0.984	0.277–3.492	0.980
Model 4						
Intercept	−2.575	0.333	−6.187	0.076	0.039–0.146	<0.001
Numbing	−0.443	0.255	3.011	0.642	0.389–1.059	0.083
Spicy	−0.221	0.187	1.401	0.802	0.556–1.156	0.237
Numbing × spicy	0.726	0.467	2.418	2.067	0.828–5.164	0.120

Model 1: unadjusted. Model 2: adjusted for age, gender, marital status, educational level, job status, average yearly income, smoking status, daily alcohol consumption, physical activity level, sleep duration, hypertension, diabetes, dyslipidemia, overweight/obesity, central obesity and poultry-aquatic dietary pattern. Model 3: excluding 381 participants with renal dysfunction, a total of 1,884 participants were included in the sensitivity analysis and adjusted for age, gender, marital status, educational level, job status, average yearly income, smoking status, daily alcohol consumption, physical activity level, sleep duration, hypertension, diabetes, dyslipidemia, overweight/obesity, central obesity and poultry-aquatic dietary pattern. Model 4: excluding 925 participants with hypertension, diabetes, dyslipidemia, a total of 1,340 participants were included in the sensitivity analysis and adjusted for age, gender, marital status, educational level, job status, average yearly income, smoking status, daily alcohol consumption, physical activity level, sleep duration, hypertension, diabetes, dyslipidemia, overweight/obesity, central obesity and poultry-aquatic dietary pattern. OR, odds ratio; CI, confidence interval.

**TABLE 7:** Additive interaction of numbing and spicy intake on hyperuricemia.

Variables	Factor 1	Factor 2	β	S.E	Wals χ^2^	*OR*	95%CI	*P*-value
	Spicy	Numbing						
Model 5								
	−	−				1.000		
	−	+	0.863	0.472	1.828	2.371	0.939–5.985	0.068
	+	−	0.997	0.457	2.181	2.710	1.106–6.641	0.029
	+	+	1.531	0.422	3.627	4.622	2.020–10.577	<0.001
RERI = 0.54 (−1.08 to 2.16)								
AP = 0.12 (−0.26 to 0.49)								
SI = 1.17 (0.65–2.11)								
Model 6								
	−	−				1.000		
	−	+	0.858	0.497	1.724	2.358	0.889–6.254	0.085
	+	−	0.518	0.487	1.065	1.679	0.646–4.363	0.287
	+	+	1.229	0.448	2.742	3.417	1.419–8.227	0.006
RERI = 0.40 (−1.11 to 1.87)								
AP = 0.11 (−0.36 to 0.58)								
SI = 1.18 (0.52–2.71)								
Model 7								
	−	−				1.000		
	–	+	0.609	0.595	1.024	1.838	0.573–5.898	0.306
	+	–	0.955	0.561	1.704	2.600	0.866–7.806	0.088
	+	+	1.548	0.515	3.009	4.703	1.751–12.902	0.003
RERI = 1.27 (−0.58 to 3.11)								
AP = 0.27 (−0.16 to 0.70)								
SI = 1.52 (0.62–3.72)								
Model 8								
	−	−				1.000		
	−	+	0.730	0.713	1.229	2.075	0.513–8.394	0.235
	+	−	0.861	0.505	1.534	2.366	0.879–6.353	0.094
	+	+	1.703	0.566	3.310	5.490	1.811–16.649	0.025
RERI = 1.37 (−0.48 to 2.11)								
AP = 0.25 (−0.12 to 0.53)								
SI = 1.32 (0.54–2.45)								

Model 1: unadjusted. Model 2: adjusted for age, gender, marital status, educational level, job status, average yearly income, smoking status, daily alcohol consumption, physical activity level, sleep duration, hypertension, diabetes, dyslipidemia, overweight/obesity, central obesity and poultry-aquatic dietary pattern. Model 3: excluding 381 participants with renal dysfunction, a total of 1,884 participants were included in the sensitivity analysis and adjusted for age, gender, marital status, educational level, job status, average yearly income, smoking status, daily alcohol consumption, physical activity level, sleep duration, hypertension, diabetes, dyslipidemia, overweight/obesity, central obesity and poultry-aquatic dietary pattern. Model 4: excluding 925 participants with hypertension, diabetes, dyslipidemia, a total of 1,340 participants were included in the sensitivity analysis and adjusted for age, gender, marital status, educational level, job status, average yearly income, smoking status, daily alcohol consumption, physical activity level, sleep duration, hypertension, diabetes, dyslipidemia, overweight/obesity, central obesity and poultry-aquatic dietary pattern. OR, odds ratio; CI, confidence interval.

### 4.8 Exploratory mediation analyses between numbing and/or spicy intake and hyperuricemia via dietary patterns

**FIGURE 6:**
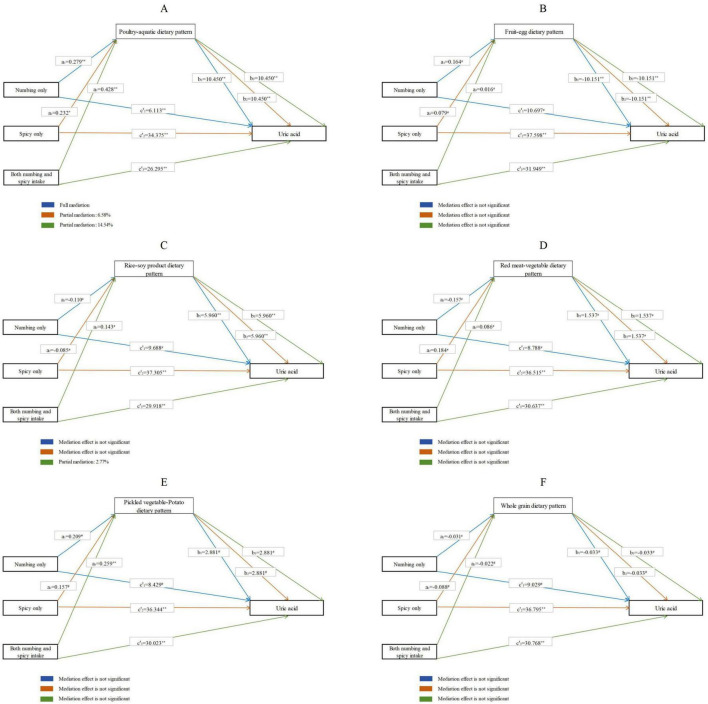
The exploratory mediation analysis between numbing/spicy intake and uric acid by whole grain dietary pattern. **(A)** Poultry-aquatic dietary pattern; **(B)** fruit-egg dietary pattern; **(C)** rice-soy product dietary pattern; **(D)** red meat-vegetable dietary pattern; **(E)** pickled vegetable-potato dietary pattern; **(F)** whole grain dietary pattern). ^#^*P*> 0.05; *P < 0.01; **P < 0.001.

Using the participants who consumed neither numbing nor spicy food as reference, we conducted exploratory mediating analysis of the six dietary patterns on the associations between numbing and/or spicy consumption and hyperuricemia (Figure 6). The poultry-aquatic dietary pattern statistically explained 6.58% of the total association of spicy-only intake and 14.54 % of the association of combined numbing and spicy intake. The rice–soy product dietary pattern statistically explained 2.77 % of the association of combined numbing and spicy intake. No significant mediation was detected for the fruit-egg dietary pattern, red meat-vegetable dietary pattern, pickled vegetable-potato dietary pattern, whole grain dietary pattern in any of the exposure-outcome pathways. The details of different mediation analyses between numbing and/or spicy intake and hyperuricemia via dietary patterns were presented in Supplementary Tables 17–22.

## 5 Discussion

The primary finding of this study is a robust and independent positive association between frequent combined intake of numbing and spicy foods and hyperuricemia (HUA) in Chinese adults. Notably, this association persisted after comprehensive adjustment for a wide range of sociodemographic, lifestyle, and metabolic confounders, as well as overall dietary patterns, highlighting that this specific culinary preference and its accompanying dietary context are associated with higher odds of HUA. he prevalence of hyperuricemia (HUA) among participants aged ≤ 60 years in this study was 13.20, which was lower than the reported national prevalence among Chinese adults aged ≥ 18 years (17.7%) (41) and among those aged 18–59 years (15.10%) (42). In contrast, it was higher than the prevalence observed in Tibetan (2.05%) (43). Disparities in HUA prevalence may be attributable to differences in age distribution, ethnicity, overall dietary patterns, genetic variants, and other population-specific characteristics, including the intake of numbing and/or spicy foods.

The consumption rates of numbing and spicy flavors among participants were 79.12% and 81.90%, respectively, indicating that these taste preferences were highly prevalent in the study region. Regarding HUA prevalence, individuals reporting consumption of neither numbing nor spicy foods had the lowest rate (3.85%), whereas those consuming only numbing foods or only spicy foods exhibited prevalence rates of 8.63 and 9.78%, respectively. In contrast, participants who consumed both numbing and spicy foods demonstrated the highest HUA prevalence (15.61%). Factor analysis identified six major dietary patterns. Participants whose diets most closely aligned with patterns characterized by higher intake of the poultry aquatic and rice soy product dietary patterns exhibited relatively higher HUA prevalence, whereas those adhering most closely to a fruit egg dietary pattern had a lower prevalence of HUA.

Multivariable logistic regression models were used to examine the association between the intake of numbing and/or spicy food and hyperuricemia. In the unadjusted model, compared with participants who consumed neither numbing nor spicy foods, the odds ratios (ORs) for those consuming numbing only, spicy only and both numbing and spicy intake were 2.361, 2.710, and 4.626, respectively. Following adjustment for confounding factors, similar associations remained significant for “numbing only” and “both numbing and spicy intake.” In contrast, the “spicy only” exposure lost its statistical association with hyperuricemia. Furthermore, the results from the RCS analysis confirmed the existence of a stable, continuous, and graded association between numbing-spicy intake and hyperuricemia. Sensitivity analysis indicated that after excluding participants with renal dysfunction, or participants with hypertension, diabetes and dyslipidemia, the association between numbing-spicy score and hyperuricemia showed a linear trend, which was inconsistent with the trend in the total population. Nevertheless, a non-linear correlation was observed among participants complicated with the above metabolic and renal diseases. This discrepancy may be attributed to the reduced sample size and decreased statistical power after exclusion in sensitivity analysis, which failed to detect the non-linear deviation effectively. It was suggested that the correlation between numbing-spicy score and hyperuricemia may show a steadily increasing linear tendency in the general population, and the non-linear characteristic was mainly driven by renal impairment and metabolic disorders.

It is important to interpret our findings within the context of local culinary practices. In southwestern China, numbing–spicy dishes (e.g., hot pot and mala xiang guo) are commonly consumed alongside purine-rich meats, aquatic products, alcohol, sugar-sweetened beverages, and high-oil/high-salt condiments. Therefore, the observed association may reflect a broader numbing–spicy-related dietary pattern rather than an independent biological effect of Sichuan pepper or chili pepper perse. Although adjustments were made for six major dietary patterns, residual confounding due to specific high-purine foods, total energy intake, fructose, sodium, and alcohol consumption could not be completely excluded. Although hyperuricemia prevalence differed significantly across dietary patterns, namely, the poultry-aquatic, rice-soy product, and fruit-egg patterns, when all six patterns were included as potential covariates in the multivariate model, only the second tertile (T2) of the fruit-egg pattern was associated with a significantly lower odds of hyperuricemia. No other dietary patterns demonstrated significant associations. Therefore, in this study, dietary patterns did not emerge as strong independent predictors of hyperuricemia, which contrasts with some previous reports (44–46).

To further examine the potential combined association of combined numbing and spicy food intake on hyperuricemia, both multiplicative and additive interaction analyses were conducted. In the multiplicative interaction models, the interaction failed to reach statistical significance. The additive interaction models demonstrated elevated joint odds ratios for combined intake; however, formal measures of additive interaction, including the relative excess risk due to interaction, attributable proportion, and synergy index were not statistically significant in any model. Therefore, we cannot conclude that numbing and spicy components exert synergistic biological effects. The higher odds ratio observed in the joint exposure group likely reflects a joint association, which may be attributable to correlated consumption patterns within the local culinary context rather than a true biological interaction. Larger prospective studies are warranted to evaluate potential interactions with adequate statistical power.

Hyperuricemia results from an imbalance between uric acid production and excretion. Excess production is often driven by dietary factors, particularly the intake of purine-rich foods and fructose. Conversely, impaired excretion is primarily determined by renal function, specifically the glomerular filtration rate and tubular secretion efficiency (47). To determine whether the observed association was influenced by broader dietary patterns, mediation analyses were conducted. These analyses suggested that the relationship between numbing and/or spicy food intake and hyperuricemia was partially statistically explained by the poultry-aquatic dietary pattern. Specifically, this pattern accounted for 6.58% of the total association of “spicy-only” intake and 14.54% of the association of combined intake of both numbing and spicy foods. Other identified dietary patterns exhibited negligible or non-significant mediating associations. These exploratory mediation analyses suggested that dietary patterns may statistically account for a small proportion of the observed association; however, longitudinal data are required to draw causal inferences regarding mediation.

To isolate the association of dietary exposure from the confounding influence of renal impairment, a sensitivity analysis was performed. After excluding 381 participants with known renal dysfunction, the analysis of the remaining 1,884 participants confirmed a persistent association. Specifically, combined consumption of numbing and spicy foods remained significantly associated with hyperuricemia. After excluding participants with hypertension, diabetes mellitus, and dyslipidemia, the similar association remained stable. These findings were further supported by the positive relationship observed in RCS analysis.

It is important to contextualize our exposure assessment within prevailing local culinary practices. In the study region, the sensory profiles of “numbing” (from Sichuan peppercorns) and “spicy” (from chili peppers) are rarely encountered in isolation in prepared foods. Signature dishes such as Sichuan cuisine, hot pot, and mala xiang guo are fundamentally defined by the combination of these two flavors. Consequently, while we statistically defined separate exposure groups for analytical purposes (e.g., “numbing only,” “spicy only”), the “both numbing and spicy” category represents the most culturally relevant and prevalent form of consumption. This investigation of the association between numbing–spicy food consumption and hyperuricemia is situated within a growing body of epidemiological evidence linking spicy food intake to cardiometabolic health (48), obesity (49), cognitive decline (50), hypertension (51). Although our study provides robust evidence for an association between frequent numbing-spicy food intake and hyperuricemia, further research is warranted to clarify biological pathways and confirm causal relationships.

Although the current understanding of the mechanisms linking numbing and/or spicy food consumption to hyperuricemia risk remains limited, the consistency of epidemiological findings underscores the need to formulate testable hypotheses. Our study provides a solid associational foundation for such inquiry. We therefore propose potential underlying pathways to explain this association. First, a key factor underlying this association may be the characteristic dietary pattern of the study region, which is predominantly meat-based and involves high consumption of numbing–spicy ingredients. In this region, meat constitutes a substantially higher proportion of numbing–spicy dishes compared with vegetables (52). Excessive consumption of high-fat meats and numbing and/or spicy foods may increase the risk of obesity, a well-established independent risk factor for hyperuricemia (51). More importantly, meat contains higher levels of purines than most other foods. In the local diet, meats such as lamb, beef, pork, and fish—particularly in purine-rich preparations such as hot pot—are commonly seasoned with numbing and/or spicy flavors (25). Excessive purine intake, particularly when combined with numbing and/or spicy foods, can directly elevate serum uric acid levels, thereby increasing the risk of hyperuricemia (53).

Second, consumption of numbing and/or spicy foods may be accompanied by increased carbohydrate intake, which could contribute to elevated BMI and weight gain. Accumulation of adipose tissue is known to stimulate excessive uric acid production (54). Unlike typical Western diets, which are predominantly meat-centered, the study region relies heavily on rice as a staple food. Numbing and/or spicy foods are believed to elevate the meal palatability through improvements in flavor, texture, color, and aroma, while concurrent carbohydrate intake may attenuate the perceived intensity of the numbing and burning sensations associated with these foods (55).

Third, condiments such as Sichuan pepper oil, chili sauce, and chili oil are widely used in local cuisine—for example, in Sichuan dishes, hot pot, and pickled foods—which may contribute to excessive fat intake and further elevate blood lipid levels (36). Elevated lipids, particularly triglycerides, can induce increased free fatty acid production, accelerate adenosine triphosphate breakdown, and promote uric acid synthesis (56). Fourth, the consumption of numbing-spicy foods is often accompanied by intake of beverages, sweets, and alcohol (57). Alcohol is known to promote uric acid synthesis and inhibit its excretion, while high consumption of sugary foods and drinks can increase obesity risk and ultimately contribute to hyperuricemia (58). In summary, the consumption of spicy and/or numbing foods, often accompanied by excessive intake of fats, carbohydrates, oils, sweets, beverages, and alcohol, may collectively increase the risk of hyperuricemia. However, the precise mechanisms underlying the relationship between numbing–spicy food consumption and hyperuricemia remain unclear. The cross-sectional design precludes causal inference, permitting only observational associations, and causality cannot be established. Prospective studies are warranted to validate and elucidate these findings.

Our findings align with, yet significantly extend, previous research. Earlier studies have often implicated heavy seasoning and specific cuisines in metabolic disorders. We move beyond this by disentangling the specific contribution of the combined numbing-spicy sensory profile and demonstrating its association with HUA independent of traditional dietary patterns. The subgroup analyses also suggested associations that appeared more pronounced in certain strata (e.g., participants aged 40–49 years, males, and those with a high school education or above). However, given the large number of subgroups examined, the lack of formal interaction testing for most comparisons, and the wide confidence intervals observed in some strata, these findings are exploratory and require confirmation in larger, adequately powered studies. Furthermore, we emphasize that future studies investigating the relationship between dietary habits and disease risk in southwestern China should not focus solely on spicy food intake. In this region, the sensory experiences of “numbing” (ma) and “spicy” (la) are intrinsically intertwined and mutually reinforcing within the culinary context. Thus, it is neither scientifically accurate nor culturally representative to examine one flavor in isolation from the other.

This study provides a novel perspective on the association between numbing-spicy food consumption and disease outcomes in Southwest China. Examining spicy food intake alone is insufficient, as relevant research cannot be conducted independently without accounting for numbing food consumption. To Our knowledge, this was the first study to explore an association between the consumption of numbing–spicy foods and hyperuricemia in southwestern China. By employing multiple analytical approaches and rigorous statistical methods, this work provides novel insights for the management of hyperuricemia. Additionally, the analysis of the influence of dietary patterns and renal function on hyperuricemia further strengthens the robustness of our findings.

Nevertheless, several limitations should be acknowledged. First, the cross-sectional design precludes definitive causal inference; reverse causality (e.g., individuals with hyperuricemia modifying taste preferences) cannot be entirely excluded. Although this is biologically less plausible given the cultural embeddedness of numbing-spicy food consumption in this region, the CMEC study is an ongoing prospective cohort, and our cross-sectional analysis serves as hypothesis-generating research that warrants confirmation in future longitudinal analyses. Second, dietary intake was assessed via questionnaire, which may be subject to recall bias and misclassification bias. For example, the intensity of numbing-spicy flavor was assessed using a standardized questionnaire based on participants’ self-reported subjective taste perception, without objective quantitative measurement of related bioactive compounds. This may inevitably lead to misclassification bias. However, the use of validated instruments and detailed assessment of food frequency and flavor intensity mitigates this concern. Our study identified an association between numbing-spicy food consumption and hyperuricemia. Given that recall bias and misclassification bias were likely non-differential, the true underlying association may be stronger.

Third, despite adjustment for six major dietary patterns, we were unable to disentangle the specific effects of numbing-spicy seasonings from those of co-consumed foods (e.g., red meat, animal organs, and seafood commonly consumed in hot pot), cooking methods (e.g., deep-frying and grilling), and accompanying beverages (e.g., alcohol and sugar-sweetened beverages) that are culturally linked to mala cuisine. Notably, numbing-spicy dishes are typically prepared with substantially higher amounts of fat and salt than non-spicy foods. Both high-fat and high-salt diets have been independently associated with the pathogenesis of hyperuricemia through mechanisms involving obesity, insulin resistance, and impaired renal uric acid excretion. Although our multivariable models adjusted for BMI, central obesity, hypertension, and overall dietary patterns, the specific contributions of fat and salt inherent to numbing-spicy food preparation could not be quantitatively separated from the sensory components themselves (i.e., Sichuan pepper and chili pepper). Therefore, we cannot exclude the possibility that the observed association was partly driven by the high-fat and high-salt culinary context of numbing–spicy food consumption rather than by the bioactive compounds in the seasonings perse. Consequently, the observed association may reflect a broader dietary pattern characterized by numbing–spicy flavor in combination with high purine, high fat, and high salt intake.

Fourth, we were unable to explicitly exclude participants receiving urate-lowering therapy (e.g., allopurinol, febuxostat, or benzbromarone), as detailed information on urate-lowering medication use was not comprehensively collected in the baseline CMEC survey. Participants undergoing such treatment would likely have lower serum uric acid levels and therefore may have been misclassified as non-hyperuricemic, potentially attenuating the observed association between numbing-spicy food intake and hyperuricemia. Although we attempted to evaluate the robustness of our findings by excluding participants with hypertension, diabetes, and dyslipidemia-groups more likely to receive comprehensive pharmacological management and the association remained stable. However, this approach could not fully account for unmeasured confounding related to urate-lowering therapy. We acknowledge that the inability to exclude these participants represents a methodological limitation. Future studies should incorporate detailed pharmaceutical assessments to determine whether the observed association persists after excluding individuals receiving urate-lowering treatment.

Fifth, dietary intake was measured at a single cross-sectional time point, which may not fully reflect long-term habitual dietary patterns. Sixth, the composite numbing–spicy score was derived from ordinal frequency categories and treated as a continuous variable in RCS analyses. This score did not reflect actual intake quantity, portion size, or concentrations of bioactive compounds (e.g., capsaicin and sanshool); rather, it served as an exploratory analytical construct. Future studies should incorporate objective biomarkers or weighed food records to improve exposure assessment. In addition, the mediation analyses were based on cross-sectional data, thereby violating the temporal ordering assumption required for causal mediation. Therefore, the proportion described as “mediated” should be interpreted as a statistical partitioning of covariance rather than evidence of a biological pathway. Furthermore, formal measures of additive interaction RERI, AP, and SIdid not reach statistical significance. Therefore, we cannot conclude combined or biological interaction between numbing and spicy components, despite a higher joint odds ratio in the combined exposure group. Finally, the study cohort was recruited from a geographically restricted population, which may limit generalizability to other ethnic or cultural groups with different culinary practices. From a public health perspective, these findings suggest that dietary guidance for hyperuricemia management should extend beyond the traditional focus on purine-rich foods. Limiting the frequency of dishes characterized by the combined intense numbing and spicy flavor—often associated with high salt, oil, and purine content—may represent a pragmatic and culturally tailored recommendation. Future prospective cohort studies are warranted to confirm temporal relationships, and mechanistic investigations should examine the direct effects of combined sanshool and capsaicin on uric acid metabolism *in vivo*.

## 6 Conclusion

This study provides the first epidemiological evidence of a positive association between numbing–spicy food consumption and hyperuricemia in adults residing in southwestern China. The observed dose–response relationship, which remained robust across multiple sensitivity analyses, suggests that higher cumulative exposure to numbing–spicy food intake is associated with a higher association with hyperuricemia. Future intervention strategies should focus on unhealthy concomitant behaviors of numbing-picy dietary habits, such as high-purine meat intake, alcohol consumption, high-oil and high-salt diet, rather than merely restricting the intake of Chinese prickly ash and chili.

## Data Availability

The original contributions presented in this study are included in this article/Supplementary material, further inquiries can be directed to the corresponding author.
