# Supplementary figures and images for: Association between numbing-spicy related dietary behavior and hyperuricemia in a Southwestern Chinese population
Source: Front Nutr. 2026 Jul 20;13:1795146. doi: 10.3389/fnut.2026.1795146 (PMC13429419; doi:10.3389/fnut.2026.1795146)

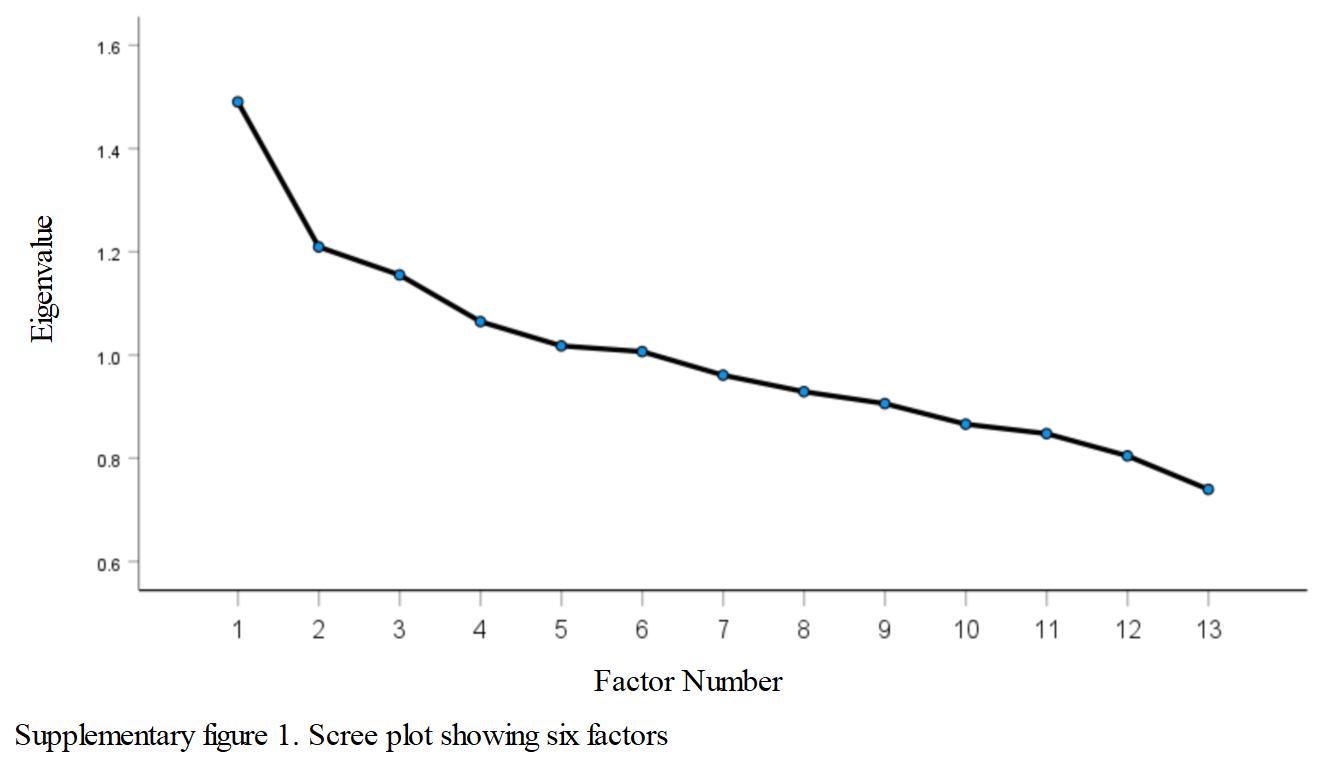

Supplement: Supplementary file 2 [file Image_1.jpeg]

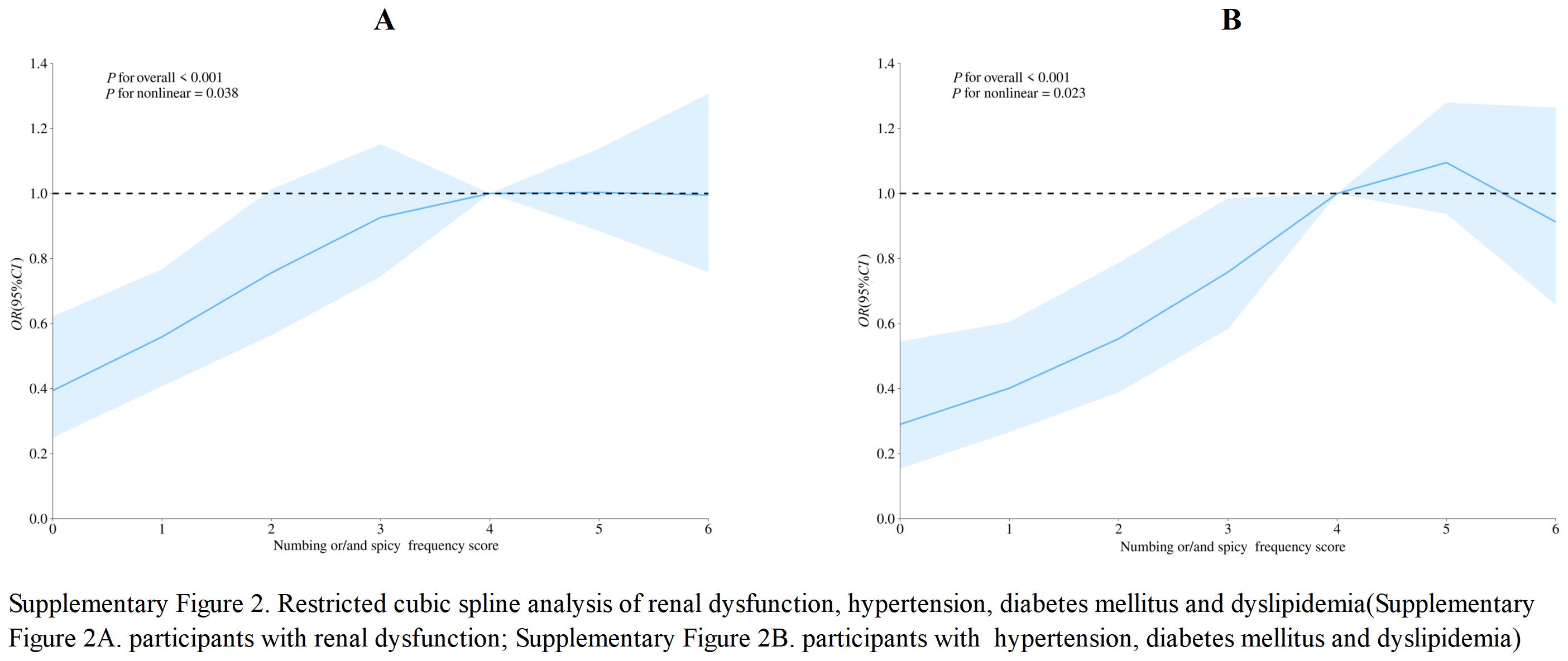

Supplement: Supplementary file 3 [file Image_2.jpeg]

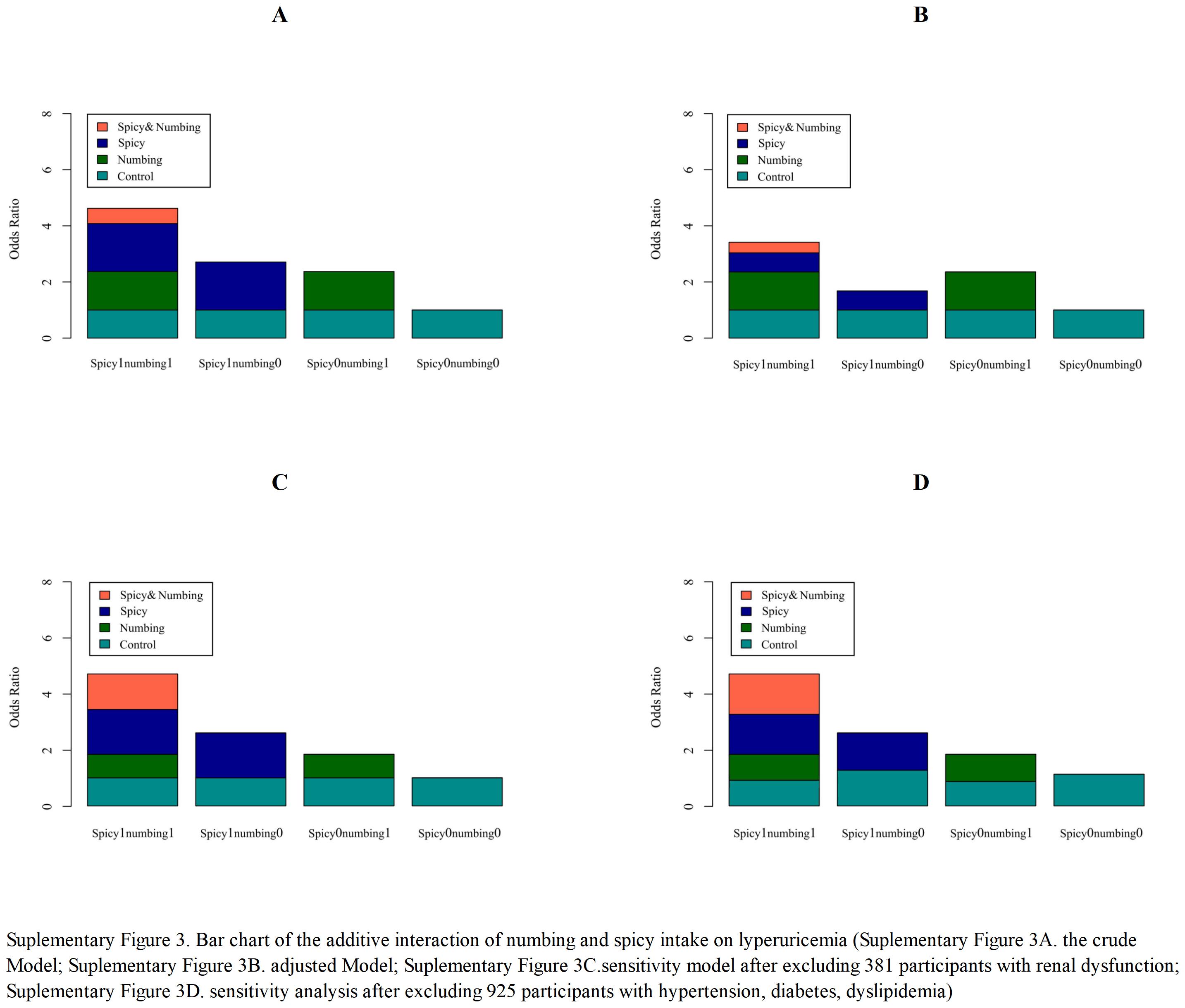

Supplement: Supplementary file 4 [file Image_3.jpeg]
